# Targeting Cancer Stem Cells—A Renewed Therapeutic Paradigm

**DOI:** 10.17925/ohr.2017.13.01.45

**Published:** 2017-05-23

**Authors:** Catherine L Amey, Antoine E Karnoub

**Affiliations:** 1 Touch Medical Media, Goring-On-Thames, UK; 2 Department of Pathology, Beth Israel Deaconess Cancer Center and Harvard Medical School, Boston, Massachusetts, US; Harvard Stem Cell Institute, Cambridge, Massachusetts, US; Broad Institute of MIT and Harvard, Cambridge, Massachusetts, US

**Keywords:** Cancer biomarker, cancer stem cell, tumor-initiating cell, microenvironment, signaling pathway, targeted therapy, radioresistance, chemoresistance

## Abstract

Metastasis is often accompanied by radio- and chemotherapeutic resistance to anticancer treatments and is the major cause of death in cancer patients. Better understanding of how cancer cells circumvent therapeutic insults and how disseminated cancer clones generate life-threatening metastases would therefore be paramount to the development of effective therapeutic approaches for clinical management of malignant disease. Mounting reports over the past two decades have provided evidence for the existence of a minor population of highly malignant cells within liquid and solid tumors, which are capable of self-renewing and of regenerating secondary growths with the heterogeneity of the primary tumors from which they derive. These cells, called tumor-initiating cells or cancer stem cells (CSCs) exhibit increased resistance to standard radio- and chemotherapies and appear to have mechanisms that enable them to evade immune surveillance. CSCs are therefore considered to be responsible for systemic residual disease after cancer therapy, as well as for disease relapse. How CSCs develop, the nature of the interactions they establish with their microenvironment, their phenotypic and functional characteristics, as well as their molecular dependencies have all taken center stage in cancer therapy. Indeed, improved understanding of CSC biology is critical to the development of important CSC-based anti-neoplastic approaches that have the potential to radically improve cancer management. Here, we summarize some of the most pertinent elements regarding CSC development and properties, and highlight some of the clinical modalities in current development as anti-CSC therapeutics.

Despite substantial advances in cancer diagnosis and treatment, the long-term survival rate for many cancer patients remains dismal.^1^ More than 90% of cancer-related mortality is ascribed to disease resurgence months or years after adjuvant therapy, either in the form of local recurrence or in the form of metastatic spread, which are typically refractory to existing treatment modalities (see Table 1).^1,2^ Novel anti-neoplastic therapeutic approaches aimed at eradicating residual relapsing disease are therefore sorely needed, but remain to be defined.

The cornerstone of current cancer management approaches relies on early detection and on chemotherapeutic and radiologic treatment of diagnosed neoplasms. Although detection methodologies have helped significantly in reducing the lethality associated with cancers such as prostate or breast neoplasms, they have had limited widespread efficacy in many others. Indeed, efforts to diagnose cancers early in their development are still hampered by serious limitations in technologies that cannot detect small tumorigenic growths or disseminated microscopic disease. Similarly, classical anti-neoplastic treatments, which target highly proliferating cancer cells, non-discriminately target bystander normal cells, such as hair follicle cells or gut-regenerating cells, causing high degree of systemic toxicity. In addition, these systemic therapies, are, to a large extent, inefficient in eradicating disseminated disease, and often result in the emergence of resistance.

The discovery that unchallenged human primary tumors harbor subpopulations of cancer cells that are distinguished from bulk populations by exclusive abilities to self-renew and generate heterogeneous secondary growths refocused attention on understanding the fundamental biology of how these cells emerge, and on identifying means to kill them. Such tumor-initiating cells (TICs), dubbed cancer stem cells (CSCs), which pre-exist already in untreated tumors, were found to be amplified in recurrent disease, and were shown to be highly malignant and with augmented tolerance to existing radio- and chemotherapeutics.^3^ Indeed, it is widely accepted that CSCs represent the root cause for metastatic dissemination and disease relapse in cancer patients. As such, the identification of effective CSC-specific therapeutics has taken center stage in the development of anti-neoplastic therapies aimed at eradicating disease relapse.^4^

The molecular underpinnings of the CSC state have been extensively reviewed over the past several years, for example, by Bandhavkar,^5^ Kuhlman et al.,^6^ and others.^7–17^ The purpose of this article is to briefly summarize and highlight some of the most pertinent concepts surrounding CSC biology, as well as current and emerging therapies targeting CSCs. Bulleted format has been used to provide a more concise presentation of these broad topics to clinicians and researchers interested in an introduction to CSC research.

New anti-neoplastic therapeutic approaches are crucial to improve long-term survival in patients with cancer. Cancer stem cells (CSCs), a subpopulation of tumor cells that self-renew and drive tumorigenesis, are emerging as therapeutic targets that can potentially revolutionize cancer patient management.

## Revival of the cancer stem cell hypothesis

- In the 1860s, Rudolf Virchow observed microscopic similarities between cancer (in this case, teratocarcinoma) and developing embryonic tissues; this led him to postulate that cancers derive from embryonic-like cells.^18^
- The concept that cancers originate from cells with stem cell characteristics was re-formulated by Julius Cohnheim.^19,20^ His theory, termed “embryonal rest hypothesis”, stated that cancers initiate from tissue-resident stem cells left over from embryogenesis, which remain dormant in such tissues until reawakened later in life to give rise to cancer.
- Cancer’s potential origin from embryonic/germinal-like progenitor cells that are inadvertently stimulated to grow uncontrollably in adult tissues was again entertained by Durante,^21^ Beard,^22^ Rippert,^23^ and Rotter.^24^
- The idea that tumors contain cancer populations with special malignant properties was re-visited by many researchers, such as Hewitt in 1953, who noted that variations in tumor-initiating potential and transplantability exist among varying inocula of sarcoma suspensions,^25^ or by McCulloch et al. in 1971,^26^ who found that tumor colony-forming cells possessed different growth characteristics than their normal counterparts, and that the so-called tumor stem cells represented a very small percentage (0.01–1%) of the whole tumor population.
- In landmark studies published in 1989, 1994, and 1997,^27–29^ John Dick’s group used a model of acute myeloid leukemia (AML) to definitively show that AML was hierarchical in nature, that cells capable of serially initiating human AML in non-obese diabetic mice with severe combined immunodeficiency disease were rare, and that they possessed self-renewal, proliferative, and differentiation capacities consistent with “true” leukemia stem cells.
- Chia-Cheng Chang and colleagues isolated two types (type I and II) of antigenically and phenotypically different normal epithelial cells from human breast tissue, and showed that only one type (type I, with luminal characteristics) is prone for transformation by SV40.^30^ Interestingly, type I could be stimulated to generate type II cells (with basal characteristics), prompting the hypothesis that cancers may originate from specialized progenitor-like cells pre-existing in solid tissues.
- Using a model in which human breast cancer cells were grown in immunocompromised mice, Al-Hajj and colleagues demonstrated that not all cancer cells within carcinomas are equally tumorigenic and that only a small subset of cells within such tumors is able to generate secondary tumors when transplanted.^31^ These observations suggested that solid cancers are, like liquid cancers,^28^ also hierarchical in nature, and harbor a small proportion of so-called tumor-initiating CSCs (also termed TICs).
- Cancer cells from several species were shown to exhibit stark activation patterns in molecular networks that otherwise function as critical regulators of embryonic, adult, and induced pluripotent stem cell homeostasis.^32–35^
- TICs/CSCs have now been identified in multiple malignancies, including multiple leukemias and various solid tumors^36^ such as lung,^37^ colon,^38^ prostate,^39^ ovarian,^40^ brain,^41^ and skin cancers.^42^
- Tumor transplantation studies in histocompatible mice suggested that CSCs can be more abundant than previously estimated, constituting as much as 10% in leukemias and lymphomas,^43^ and as much as 25% in melanomas.^44^
- The proportions of CSCs within tumors correlate positively with poor prognosis.^45^

A better understanding of CSC biology will not only lead to conceptual advances in understanding tumour etiology, but will also catalyze the development of important CSC-based anti-neoplastic approaches that have the potential to radically improve cancer management.

## Characteristics of cancer stem cells

- Normal self-renewing adult tissue stem cells give rise to progenitor cells that are often termed transit-amplifying cells which, in turn, divide and proliferate to engender more differentiated cells with restricted proliferating and clonogenic potentials. This hierarchical system calls upon stem cells (which sit at the top of the pyramid) to expand when more differentiated cells (laying at the bottom of the tissue pyramid) are depleted.
- The self-renewing ability of these stem cells ensures their continued presence within tissues and the balance between stem and differentiated cells ascertains tissue homeostasis.
- Normal stem cells are in constant interactions with their microenvironment, or niche, which tightly regulates stem cell state maintenance while controlling the expansion of the stem cell compartment.
- Tumors are formed of heterogeneous cancer cells that are organized in a hierarchy similar to that of normal tissues, and contain CSCs that share several characteristics with normal stem cells.
- The 2006 American Association for Cancer Research Workshop on Cancer Stem Cells defined a CSC as “a cell within a tumor that possesses the capacity to self-renew and to cause the heterogeneous lineages of cancer cells that comprise the tumor.”^46^
- In this model, each tumor contains a sub-population of cells (the CSCs) that are able to divide asymmetrically in order to self-renew and give rise to a phenotypically distinct daughter cell. CSCs are thus considered to be the source of all aggressive plastic cancer cells present in a malignant tumor.^2^
- Even within the CSC compartment, CSCs are heterogeneous, with some possessing short-term repopulating potential while others exhibit longer-term repopulation abilities.^47^
- By virtue of their ability to initiate new tumors, CSCs are thought to represent the cellular seeds responsible for tumor recurrence/relapse and the ones that give rise to distant metastases.^2^
- CSCs shuttle between quiescent and active states, and are characterized by their generally slow proliferating rates.^48^
- CSCs occupy specialized niches or tumor microenvironments (TMEs) that maintain stem cell-like properties of such cells via interactions mediated by the extracellular matrix (ECM) and activation of various signal transduction pathways.^49,50^
- CSCs exhibit increased drug resistance activities, attributes that are mediated by multiple mechanisms that include, among others, cell dormancy, hypoxia, enhanced activity of DNA repair enzymes, higher expression of drug efflux transporters (e.g., expression of the ATP-binding cassette [ABC] transporters, such as ABCB1 and ABCG2),^51,52^ and an elevated expression of anti-apoptotic proteins.^4^
- As such, CSCs exhibit increased resistance to standard radio- and chemotherapies.^53–56^
- Multiple stemness pathways are deregulated in CSCs, which include Wnt, tumor growth factor-beta (TGF-β), signal transducer and activator of transcription (STAT), and Hippo-yes associcated protein (YAP)/transcriptional co-activator with PDZ-binding motif (TAZ) (see Table 2).^57^
- CSCs also evade immune surveillance, which relies on innate and adaptive immune cells recognizing traits of malignant transformation. This occurs via multiple mechanisms that include the following:
  - CSCs reduce (and even inhibit) the expression of tumor-associated antigens (TAAs), which limits their recognition and elimination by cytotoxic T cells (CTLs).^58^
  - CSCs downregulate the expression of MHC class I molecules.^59–61^
  - CSCs express CD95 and CD95 ligand, which in addition to its autocrine role in promoting CSC state, can kill neighboring CTLs.^62,63^
  - CSCs express “don’t eat me” signals, such as CD47, which prevents their phagocytosis by macrophages.^64,65^
  - CSCs may utilize the programmed death-1 (PD-1)/PD ligand-1 (PD-L1) system to inhibit the immune system.^66,67^ PD-L1 binds its receptor, PD-1, which is found on multiple immune system cells such as CTLs, natural killer (NK), and dendritic cells. PD-L1/PD-1 signaling is thought to play a major role in inhibiting lymphocyte proliferation, thereby enabling the tumor cells to evade immune surveillance.
  - In addition, CSCs secrete immunosuppressive chemokines and cytokines that can inhibit CTL functions (e.g., TGF-β and interleukin 10 [IL-10]).^11,68^
  - CSCs may regulate the recruitment of T-reg cells,^11^ which have diverse immune modulatory functions in cancer.^69^

The plasticity of CSCs, their ability to resist standard radiotherapies and chemotherapies, and their capacity to evade immune surveillence position them as key determinants of cancer malignancy.

## Identifying cancer stem cells

- Surface markers can be used to differentiate CSCs from the tumor bulk. Partial phenotypes of CSC markers, organized according to cancer types, are presented in Table 3.^36^
- Such antigenic marker profiles have enabled the isolation of human CSCs from heterogeneous tumors using fluorescence-activated cell sorting^28^ or antibodies conjugated to magnetic beads.^70^
- Using these approaches, as well as efflux-based dye labelling (e.g., side population labelling), CSCs have been isolated from multiple solid and liquid tumors, including leukemias, lung, colon, prostate, ovarian, brain, and skin cancers,^36–42^ to name a few. For example:
  - Leukemia stem cells (LSC) display a CD34^+^CD38^−^ phenotype;^28^ the “true” CSCs can also be CD123^+^ or IL3-alpha^+^.^71^
  - Certain breast CSCs have a CD44^+^CD24^−^ phenotype.^9^
  - Brain CSCs are also CD133^+^, similar to brain stem and progenitor cells.^72^
  - Ovarian CSCs are CD117^+^ and CD133^+^.^73^
- Three major *in vitro* and *in vivo* functional assays have been used to characterize CSCs:
  - *In vitro* colony-forming assay,^74^ which relies on the ability of CSC-like cells to form colonies (or tumorspheres) under serial passages in three-dimensional cultures.
  - Limiting dilution analyses (LDA), which test the ability of CSCs to initiate tumors at low cell densities in immunodeficient mice.^75^
  - Transplantability assays, which test the abilities of CSC-like cells to regenerate the tumor bulk once transplanted into animals, and the transplantability of these cells from one animal into another.^28^

Surface marker analyses have been instrumental in the identification of CSCs from bulk tumors, but it is important to note that such markers are not universal, even within the same tumor type, and that they result in the underestimation of CSC content in a certain tissue. In addition, the mechanical and/or enzymatic disruption of tumor tissues prior to the antibody-mediated isolation of putative CSCs from such preparations stands to alter the antigenic profiles of CSCs. Similarly, it is also important to note that the estimate of CSC content in tumors using the LDA approach is complicated by the fact that tumor-initiation efficiency in recipient mice depends on the level of immune deficiency present in these recipient animals.^27^

## Cancer stem cell genesis

### Cancer stem cell genesis models

**Figure 1:**
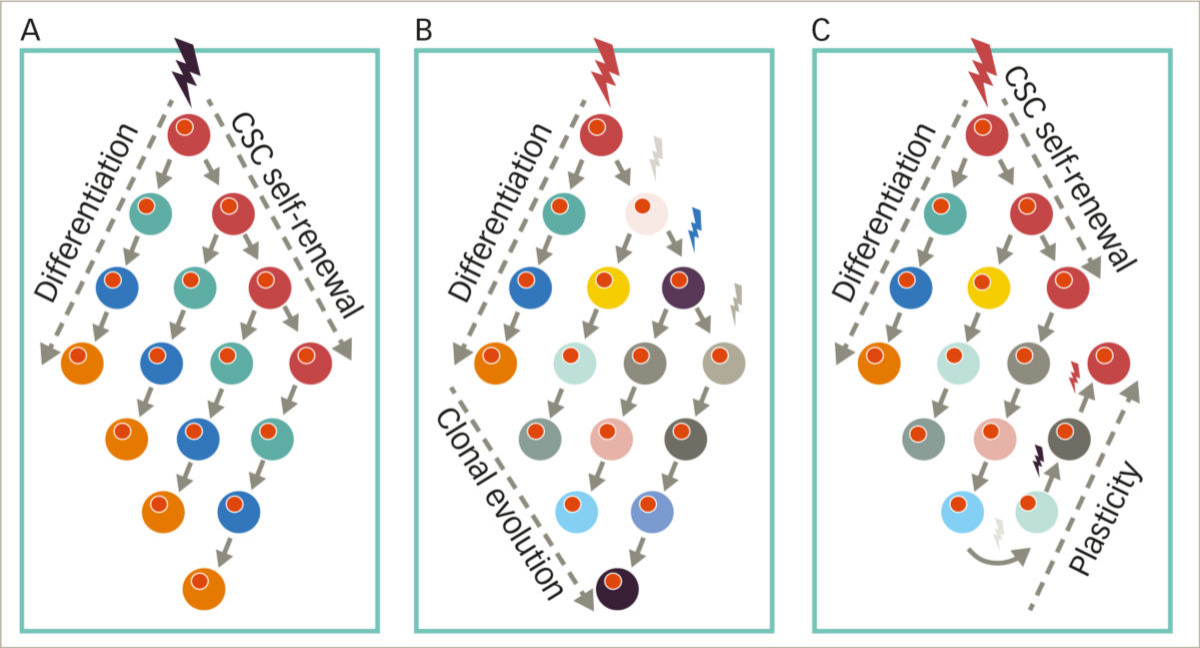
Cancer stem cell models Hierarchical model (A), stochastic (B), and plasticity model (C).

- In the hierarchical model, cancer is thought to initiate from stem/progenitor cells with intrinsic capacities for self-generation. Accordingly, tumors are viewed as hierarchical pyramids whereby, at the peak, CSCs can self-renew while generating non-CSC differentiated progeny, which form the rest/base of the pyramid, or the tumor bulk.
- Alternatively, the stochastic or clonal evolution model (CE model) posits that a variety of established tumor cells can contribute to generating TICs, in varying degrees, via both intrinsic (e.g., oncogenic lesions) and extrinsic (e.g., microenvironmental) factors such as TGF-β76 or hypoxia.^77,78^
- Reconciling these two seemingly opposing theories is the CSC plasticity model, in which the CSC state is a dynamic state that can be gained or lost based on external cues and the innate propensity of the tumor cells to plasticity. Cures according to this model and the stochastic models can only be achieved when treatments result in the death of sufficient numbers of tumor cell populations, hindering possibilities for tumor CSC regeneration and/or expansion (see Figure 1).^79^
- Of note, Tomasetti and Vogelstein^80^ demonstrated, through statistical analysis, that the lifetime risk of different types of cancer is correlated strongly with the number of divisions of the normal self-renewing cells that maintain the homeostasis of these tissues. Therefore, variation in cancer risk among tissues can be explained by the number of stem cell divisions such tissues undergo, suggesting that tissue-resident stem cells are the origin of tumors or that their high turnover predisposes them to oncogenic mutations.

### How cancer stem cells arise

- There are several proposed hypotheses to explain how CSCs arise, including the following:
  - Normal stem cells lose the constraints that limit their self-renewal, and accumulate oncogenic lesions while proliferating.^78^ In this view, normal stem cells themselves give rise to CSCs.^81^
  - More differentiated transit-amplifying cells may acquire oncogenic mutations that cause the aberrant activation of their self-renewal pathways,^78^ leading to their uncontrolled proliferation while inhibiting their terminal differentiation.^82^
  - Oncogenic mutations acquired by differentiated cells lead to their dedifferentiation towards more stem-like cells.^82–83^
  - Mechanical- and/or chemical-mediated disruption of intercellular communication (e.g., via disruption of gap junctions), postulated a long time ago to be one of the main features of tumor tissues,^84^ can increase the propensity for CSC generation.^78^
  - Fusion of normal stem cells and cancer cells, although rare, can also give rise to CSC-like populations within tumors.^85^
- The TME in CSC development:
  - The TME contains a variety of cells, including inflammatory and fibroblastic cells, such cancer-associated fibroblasts and mesenchymal stem/stromal cells. These cells affect tumor growth, development and progression in early tumorigenesis and influence the microenvironment to sustain tumor growth as well as secondary tumor formation (metastasis) in other tissues.^94^
  - Co-injection of stromal cells with cancer cells facilitates tumor formation in immune-deficient mice.^95–97^ This suggests that TICs, hence CSC-like cells, are indeed aided by microenvironmental factors produced by surrounding stromal cells.
  - The TME may facilitate the plethora of CSC characteristics that allow a tumor cell to accumulate enough epigenetic and genetic changes over time to become highly malignant.^98^ Importantly, CSC-niche cross-talk may occur whereby the niche might not only regulate CSC traits, but may directly provide CSC traits to non-CSCs.^99^
  - CSCs may foster a favorable niche by promoting the formation of a reactive TME.^50^ For example, secretion of TGF-β2/TGF-β3 from breast cancer cells that disseminated to the lung has been shown to induce stromal fibroblast expression of periostin (POSTN), which is a component of the ECM. In turn, TME-derived POSTN induces recruitment of Wnt ligands, which increase Wnt signaling in the metastasis-initiating CSCs.^100^
- Hypoxia in CSC development:
  - Hypoxia actively induces and maintains CSC phenotype,^98,101,102^ for example, in breast cancer^10^ and glioblastoma.^103^
  - The effects of hypoxia on CSCs seem to be primarily mediated by hypoxia inducible factors (HIFs), particularly HIF2α.^101^
  - Hypoxia induces a spectrum of changes that may contribute to malignancy including the selection of apoptosis-resistant clones^104^ and promotion of tumor invasion and metastasis.^105^
  - HIF inhibitors can block chemotherapy-induced enrichment of CSCs, suggesting that HIFs play critical functional roles in CSC biology, and that their inhibition may increase survival in patients.^10^
- The epithelial-mesenchymal transition (EMT) in CSC development:
  - The EMT is a crucial developmental program often activated during cancer invasion and metastasis. During this process, polarized epithelial cells are converted to mesenchymal cell-like migratory tumor cells.^106,107^
  - Transcription factors that regulate migration and EMT in embryonic tissues regulate EMT in cancer cells.^108^
  - Several findings have indicated a direct link between the EMT and gain of epithelial stem-cell properties.^109,110^
  - Many EMT transcription factors, including zinc finger protein SNAI1 (also referred to as Snail), zinc finger E-box-binding (ZEB) and basic helix-loop-helix transcription factors,^108^ have been shown to regulate CSC phenotype and function in breast, pancreatic, and colorectal tumors.^99^
  - EMT transcription factor pathways promote additional malignancy traits ascribed to CSCs, including therapy resistance and anti-apoptosis.^111–113^
- Stiffness/tensegrity in CSC development:
  - Growing evidence indicates that physical constraints, such as stiffness and porosity of the ECM, can influence tumor behavior as a whole.
  - For example, matrix stiffness increased the proportion of human head and neck squamous cell carcinoma (HNSCC) TICs, concomitant with inducing higher tumorigenicity and metastasis.^114^
  - High matrix stiffness promotes proliferation and increased chemotherapeutic resistance in hepatocellular carcinoma cells.^115^
- Fusion:
  - One other possible but controversial potential mechanism for the generation of CSCs is through fusion between stem cells and differentiated cells.^85^

Evidence indicates that CSCs may be generated and maintained by a plethora of mechanisms that can depend on genetic and epigenetic properties intrinsic to cancer cells, as well as on extrinsic stimuli emanating from the TME or niche. What is clear is that some CSCs are “born” while others are “made” by the tumor milieu. Although these notions can seem contradictory, it is attractive to postulate that both operate in tumors, and that the extent to which one mechanism is empahsized over the other depends on the stage of tumor progression.

## Signaling networks in the makeup of the cancer stem cell state

- The ability of CSCs to retain their properties is determined by an array of signaling networks that are responsive to intrinsic and extrinsic stimuli.
- Pathway elements that play a role in the control of self-renewal and differentiation of CSCs include:
  - Phosphatidylinositol-3-kinase (PI3K)/Akt and the mammalian target of rapamycin (mTOR) pathway activation are critical regulators of cell proliferation and survival. Increasing reports have also underscored the importance of PI3K in regulating the CSC state.^116,117^
  - The Janus-activated kinase (JAK) signaling pathway has been implicated in tumorigenesis through STAT-3 activation.^118^ Blockade of JAK-STAT has been shown to inhibit tumor initiation and clonogenic recovery of prostate CSCs, substantiating a role for this pathway in tumor initiation.^119^
  - Nuclear factor-kappa B (NF-κB) is an inducible transcription factor that affects the expression of several apoptosis-related proteins and cell cycle regulatory components. It has been shown to be upregulated in many cancers, and has been implicated in CSC genesis, as well as promoting invasion and metastasis.^120^
  - Mammals express four transmembrane Notch receptors (Notch-1, −2, −3, and −4)^121^ and five canonical transmembrane ligands (Delta-like [DLL] 1, DLL3, DLL 4, Jagged-1, and Jagged-2).^122–125^ Notch signaling is an evolutionarily conserved pathway involved in the control of cell fate, with roles in carcinogenesis, tumor angiogenesis, and EMT.^126^ Overexpression of Notch signaling components has been reported to promote self-renewal of CSCs in a variety of malignancies and is involved in the interaction between the tumor and the stroma in both primary and metastatic tumors.^127,128^
  - Hedgehog is a key morphogen regulating embryonic development and tissue repair. It has been implicated in the maintenance of the CSC phenotype.^129^ Interestingly, it regulates the expression of the ATP-binding cassette sub-family G member 2 and multi-drug resistance genes, implying involvement of Hedgehog overexpression in the development of the chemoresistance characteristic of CSCs.^130–133^
  - Wingless/integration (Wnt) signal transduction pathways play an important role in cell fate specification, cell proliferation, and cell migration. When aberrantly expressed, Wnt contributes to the tumorigenic potential of CSCs.^134,135^
  - The YAP and TAZ are the major downstream effectors of the Hippo pathway, which regulates tissue homeostasis, organ size, regeneration, and tumorigenesis.^136^ YAP and TAZ are regulated by soluble extracellular factors, cell–cell adhesion, and mechanotransduction, and such regulation appears to be disrupted in cancer.^137–140^
  - Enhanced nuclear accumulation of YAP1, due to upstream inhibition of Hippo signaling, has been shown to increase epidermal squamous cell carcinoma spheroid formation, invasion, and migration.^141^ Hippo pathway inhibition has also been shown to be a requirement for the enhanced migratory and invasiveness properties of breast cancer cells.^142^ In breast CSC, TAZ activity sustains self-renewal and tumor initiation.^143^
  - Focal adhesion kinase (FAK) is a non-receptor tyrosine kinase, overexpressed in cancer, with roles in adhesion, survival, motility, metastasis, angiogenesis, and lymphangiogenesis. Involvement of FAK in CSC functions have also been reported.^144,145^
  - Homeobox (HOX) genes are an evolutionarily highly conserved family of proteins with demonstrated regulatory roles in cell fate determination.^123^ Members of the HOX gene family have been implicated in tumor development and progression.^146^ Cross talk between HOX and other CSC-regulating mediators, such as sonic hedgehog, Wnt, and Notch signaling pathways^147^ suggest a central role for HOX in CSC homeostasis, and underscore its potential as a credible therapeutic target in the context of CSC-based therapy.
  - EMT transcription factors (e.g., SNAIL, TWIST, Zinc finger protein SNAI2/SLUG) are induced during carcinoma progression^148^ and they exert critical determining roles in the promotion/genesis and in the maintenance of CSC traits.^149^
  - Bmi-1, a polycomb family proto-oncogene, is required for the self-renewal of diverse adult stem cells. It promotes stem cell self-renewal partly by repressing the expression of the tumor suppressor genes Ink4a and Arf.^150^ Roles for Bmi in CSC regulation have been described in breast cancer,^151^ leukemias,^152^ prostate cancer,^153^ pancreatic cancer,^154^ and in many other malignancies.
  - Various protein kinase C isoforms have been implicated in the renewal of normal and CSCs.^155^ For example, PKC-iota (PKCi) was shown to promote CSC traits in ovarian carcinoma cells.^156^ Similarly, atypical PKC (aPKC) was shown to promote CSC-like EMT traits in a model of prostate cancer.^157,158^

The CSC state is maintained by the interplay of various signaling pathways. The complexity of these networks ensures redundancy in the maintenance of cancer cell stemness, and stands to frustrate therapeutic efforts aimed at inhibiting single molecular nodes. Renewed CSC-targeted therapies with clinical applicability therefore have to encompass the simultaneous inhibition of several critical targets to achieve clinical efficacy.

## Cancer stem cells at the crossroads of metastasis and therapy resistance

### Metastasis

- CSCs have been hypothesized to contribute directly to metastasis. Indeed, secondary tumors are initiated by cancer cells with the capacities of making new growths, drawing strong parallels between the CSC state and the metastatic state.^159,160^
- Primary tumors derived from the implantation of CSCs isolated by the putative stem cell markers CD44^+^ and CD24^−/low^ generate abundant lung metastases,^161^ suggesting that TICs within primary tumors can generate metastatic CSCs.
- There are strong functional links between CSC markers and metastatic phenotypes. For example, CSC-associated CD44 is a homing and adhesion marker, and it has demonstrated activities in enhancing metastatic capabilities.^31,162^
- Although CSC preponderance in primary tumors has been correlated with an increased incidence of metastasis, a causal relationship between the primary tumor CSCs and the cells-of-origin of distant metastases has not been proven.^163^

### Therapy resistance

- Drug resistance invariably develops in most cancer patients on therapeutic regimens, limiting outcome and long-term survival.^164,165^
- Better understanding of how drug-resistance develops during initial tumor response and regression will lead to the development of more effective therapeutic modalities.
- For example, therapeutic inhibition of oncogenic drivers in drug-sensitive cancer cells has been shown to induce secretome changes that, paradoxically, establish a TME that supports the expansion of drug-resistant clones.^166^
- Mounting evidence suggests that therapy-resistant clones share a great deal of similarities with CSCs.^167^
- Indeed, evidence from several cancers indicates that CSCs are highly resistant to ionizing radiation,^168–176^ as well as chemotherapy.^171,177^
- Underlying mechanisms through which CSCs resist therapy include:
  - high free radical scavenger status;^168,178^
  - cell-type specific fuctuations in proteasome activity;^179,180^
  - expression of the *ABCB5* multi-drug resistance protein;^181^
  - enhanced DNA repair capacities and the ability to maintain low ROS content;^182^
  - upregulation of oncogenic pathways;^183^
  - upregulation of anti-apoptotic nodes, such as survivin;^184^
  - upregulation of stem-cell-regulating master genes, such as Oct4.^185–188^

CSCs have been strongly linked to metastatic dissemination and therapeutic resistance. Prevention of tumor relapse therefore depends on eliminating CSCs, derailing their development under classical therapeutic management, or inhibiting the reawakening of disseminated tumor cells from dormancy.

## Therapeutic opportunities for cancer stem cell-based therapies

- Resistance of CSCs to current chemotherapeutics and radiotherapies are major factors contributing to cancer recurrence.^4^
- CSCs have been shown to be essential for tumor pathogenesis and CSC targeting has proven to be effective in suppressing tumor development in a number of pre-clinical proof-of-principle experiments.^181,189^
- CSC-based therapeutics therefore represent an attractive route towards developing cancer cures, and are aimed at interfering with the functions of surface markers, drug efflux channels, stemness pathways, epigenetic regulators, as well as key oncogenic signaling nodes that are essential for CSC homeostasis. Differentiation therapies as well as immunotherapeutics are also being developed with CSCs as their focused targets.
- Complicating these efforts, however, are the facts that not all CSCs express specific and exclusive markers,^16^ and that they may have dynamic phenotypes and genotypes,^190^ raising the difficulty in developing effective CSC-targeted therapies.
- Nevertheless, many novel and innovative approaches are in different stages of drug development and clinical testing, and some targets/pathways are enumerated here for illustration purposes.
  - **CD133.** Interest has grown in the use of monoclonal antibodies to target CSC surface markers. One example of this is CD133 (prominin-1), a cell surface glycoprotein that is expressed widely in solid tumors (see Table 3), and which has been associated with drug-resistant phenotypes and poor prognosis.^191^ CD133-positivity marks CSCs in a variety of tumors, including colon,^192^ brain,^72,193,194^ and lung.^195^ Anti-CD133 cell therapy has been tested and shown to reduce the proliferative capacities of TICs.^196^
  - **PI3K/mTOR.** The PI3K/mTOR signaling pathway, a key regulator in cancer progression and CSC survival, is targeted by VS-5584 (Verastem), which is an inhibitor of PI3K, mTORC1, and mTORC2 that preferentially destroys CSCs. Verastem is currently conducting a phase I trial of VS-5584 in patients with advanced cancer (ClinicalTrials.gov identifier NCT01991938). Biomarkers of response to VS-5584 will also be assessed in archival tumor tissue, tumor biopsies (in consenting subjects), and blood samples.
  - **ABC.** The ABC-driven efflux transporters are largely responsible for chemoresistance,^197^ and all the mechanisms involved in ATP transporter modulation may be potential therapeutic targets. Numerous members of ABC transporters have been described, although only a few are known to be expressed in human CSCs: multidrug resistance 1 (MDR1) or P-glycoprotein (Pgp)/ABCB1, multidrug resistance protein 1 (MRP1/ABCB1), breast cancer resistance protein (9BCRP), and the melanoma-associated, chemoresistance mediator, ABCB5. Schatton et al.^181^ identified a subpopulation enriched for human malignant melanoma-initiating cells (MMICs) that was defined by expression of *ABCB5* and showed that specific targeting of this population inhibits tumor growth. Drugs targeting ABC transporters have had limited efficacy in clinical trials so far; however, they may prove more effective if used in combination with other anticancer agents that target CSCs.^191^
  - **Notch.** As one of the most intensely studied potential therapeutic targets, several inhibitors of the Notch pathway are being developed.^126^ These include monoclonal antibodies targeted against Notch receptors or Notch ligands and blocking peptides and inhibitors of the Notch inhibitor γ-secretase (GSIs). Demcizumab is a humanized immunoglobulin G2 antibody that binds to Delta-Like Ligand 4 (Drosophila, DLL4), and is being studied in an ongoing phase 1b dose escalation study in combination with pemetrexed and carboplatin in chemotherapy-naïve stage IIIb/IV non-squamous non-small cell lung cancer.^198^
  - **Focal adhesion kinase (FAK).** FAK inhibitors under study include defactinib (VS-6063) and VS-4718, NVP-TAE-226, pyrrolopyrimidines, and PND-1186.^145^ VS-4718 is currently being investigated in subjects with metastatic non-hematologic malignancies (NCT 01849744) (Table 3).
  - **Wnt.** Several agents have been developed for targeting of this pathway, e.g., OMP-54F28, rofecoxib; PRI-724, CWP232291; and monoclonal antibody against frizzled receptors, vanituctumab.^199^
  - **Nanog.** Amcasertib (BBI503) is an orally administered investigational agent designed to inhibit Nanog and other cancer stem cell pathways by targeting kinases.^200^ A dose escalation study (n=26) established the recommended dose of amcasertib at 300 mg/day. Prolonged disease stabilization was reported in several heavily pre-treated patients and phase II trials are ongoing.
  - **STAT3.** STAT3 activity has been shown to regulate self-renewal of CSCs.^201,202^ In phase Ib/II trials, the STAT3 inhibitor, napabucasin (BBI608), has shown promising anticancer activity when used in combination with other agents across advanced, pretreated and untreated metastatic pancreatic cancers, as well as advanced, pretreated colorectal cancer.^203–205^ Napabucasin plus weekly paclitaxel in the treatment of gastric and gastroesophageal junction cancer is being studied in the phase III BRIGHTER trial (NCT02178956). The final data collection date for primary outcome measure for the BRIGHTER Trial is August 2017. Napabucasin is also being studied (phase III) in colorectal cancer (CanStem303C, NCT02753127) and pancreatic cancer (CanStem111P, NCT02993731).
  - **Hedgehog.** There is compelling evidence to suggest that inhibition of Hedgehog signaling in CSCs results in loss of stemness, as supported by a reduction in clonogenicity and pluripotency markers, thereby limiting the characteristics that would otherwise support chemoresistance. Targeting of CSCs and tumor bulk with Hedgehog inhibitors and conventional chemotherapeutics and/or radiation is thus a potential approach to prevent tumor relapse and improve patient outcomes.^206^
  - **CXCL12.** CXCL12, also known as stromal-derived factor-1, is a chemokine that binds its receptor, CXCR4, and is involved in migration, invasion and survival of normal and malignant cells.^207^ Involvement of CXCL12 in regulating several aspects of CSC biology has been documented,^208^ and its inhibitors show promise in experimental models.^209^
  - **CD47.** CD47 represents a “don’t eat me signal” in CSCs and plays a significant role in inhibiting their phagocytosis; CD47 inhibition therefore could enhance immune cell (e.g., macrophage) -mediated elimination of CSCs.^210–212^ Leukemia stem cell function in murine xenotransplantation models of AML has been reported to depend on inhibition of macrophages via the immunoglobulin superfamily receptor, SIRPα.^213^ Disrupting SIRPα signaling in macrophages by preventing engagement with its ligand, CD47, eliminated AML stem cells in the xenografts. Also, blocking CD47 signaling has been shown to promote engulfment of pancreatic neuroendocrine tumor cells by macrophages *in vitro* and inhibited xenograft tumor growth, preventing metastases, and prolonging survival *in vivo*.^214^
  - **Interleukin-4 (IL-4).** IL-4-mediated drug resistance has been demonstrated in colon CSCs,^215^ providing the rationale for anti-IL-4 antibody or IL-4 receptor-alpha antagonists as anti-tumor therapies.
  - **Proteasome.** Glioma stem cell (GSCs) have been shown to be 1,000-fold more sensitive to proteasomal inhibition compared with differentiated controls,^216^ which provides a further new potential strategy for targeting stem cells versus bulk cancer cells.
  - **Tenascin.** Emerging evidence on additional mechanisms by which CSCs evade immune surveillance may enable the development of novel therapeutics. For example, CSCs derived from either prostate draining lymph nodes (PDLN) or mice harboring oncogene-driven prostate intraepithelial neoplasia (mPIN) use the ECM protein, Tenascin-C, to arrest T-cell activation by interacting with α5β1 and blocking reorganization of actin-based cytoskeleton.^217^

Translational strategies aimed at targeting CSCs are well underway, with many in advanced pre-clinical stages and others in bona fide clinical trials. Considering that the mulitple and often redundant number of pathways that uphold the CSC state, it is likely that combinatorial use of the aforementioned agents, with or without chemo- or radiotherapies, would prove most advantageous in disease management. It is also hoped that such CSC-directed approaches will reduce the toxicity associated with traditional cancer therapies, promoting longer patient survival, while ensuring appropriate quality of life metrics.

## Concluding remarks

CSCs mediate tumor metastasis and, by their increased resistance to chemotherapy and radiation therapy, contribute to treatment failures and disease relapse.^218^ CSC-targeted therapies therefore have the potential to achieve higher efficacy and remission rates than standard regimens, and stand to revolutionize cancer management.

With this optimistic view comes the inherent plasticity of CSCs, which enables them to transition between CSC and non-CSC states as cells exhibit both functional and phenotypic heterogeneity.^219^ In this scenario, targeting specific CSC populations may ultimately prove futile. Rather, the potential of CSC-directed therapies may be more realized in conjunction with existing chemo- and radiotherapies (or even immunotherapies). Indeed, comprehensive combination strategies have been suggested to improve cancer treatments, which rely on, for example, a ligand targeting CSC, an ABC transporter inhibitor to overcome drug resistance, coupled with an imaging agent to facilitate tumor response diagnosis.^36^

Ultimately, therapies that target CSCs are hoped to emerge as critical components of avant-garde effective clinical strategies, particularly in malignancies that continue to exhibit high mortality rates, such as pancreatic, brain, and lung cancers. The field has come a long way, and as translational research into CSC biology is gaining pace, the clinical applications of such advances appear to be increasingly tenable.

## Figures and Tables

**Table 1:** Five-year cancer survival statistics

Site	5-year survival (%)
Bladder	77
Breast	89
Colorectal	65
Kidney	73
Liver	17
Lung—non-small cell	17
Lymphoma—non-Hodgkin	70
Melanoma	92
Oral and oropharyngeal cancer	63
Ovarian, fallopian tube, and peritoneal cancer	58
Pancreatic	7
Prostate	99
Thyroid	98
Uterine	82

Data sourced from: www.cancer.net

**Table 2:** Examples of targeted stemness pathways under clinical investigation^57^

Tumor type	Agent	Target	Phase
Breast	Vantictumab^a^ with SOC chemotherapy	Wnt	I
CLL, relapsed/refractory	Duvelisib^b^	PI3K Delta/PI3K Gamma	III
CLL, first line, younger patients	Duvelisib^b^ with FCR	PI3K Delta/PI3K Gamma	I/Ib
Colorectal	Napabucasin^c^	STAT	II/III
Gastric	Napabucasin^c^	STAT	III
Gastrointestinal	Napabucasin^c^	STAT	II/III
	Amcasertib^d^	Broad spectrum, incl. Nanog	II
Glioblastoma	Napabucasin^c^	STAT	II
Hematologic malignancies	Napabucasin^c^	STAT	I
	Bronticuzumab^e^	Notch	Ia
Hepatobilary cancer	Amcasertib^d^	Broad spectrum, incl. Nanog	II
Hepatocellular carcinoma	Napabucasin^c^	STAT	I
	Amcasertib^d^	Broad spectrum, incl. Nanog	I/II
Mesothelioma	Napabucasin^c^	STAT	II
	Defactinib^f^	FAK/PYK2	I/Ib
iNHL, refractory	Duvelisib^b^	PI3K Delta/PI3K Gamma	II
Non-small cell lung cancer	Defactinib^f^ with pembrolizumab		
	FAK/PYK2	I/Ib	
	Demcizumab^g^ plus SOC chemotherapy	Notch	II
Small cell lung cancer	Tarextumab^i^	Notch	II
	Napabucasin^c^	STAT	III
Ovarian	Amcasertib^d^	Broad spectrum, incl. Nanog	II
	Defactinib^f^	FAK/PYK2	I/Ib
	Demcizumab^g^ and paclitaxel	Notch	Ib/II
	Ipafricept^h^ with carboplatin/paclitaxel	Wnt	Ib
Pancreatic	Defactinib^f^ with pembrolizumab + demcitabine	FAK/PYK2	I/Ib
	Napabucasin^c^	STAT signaling cascade	III
	Defactinib^f^		
	FAK/PYK2	I/Ib	
	Vantictumab^a^ with gemcitabine and abraxane	Wnt	Ib
	Ipafricept^h^ with gemcitabine/nab-paclitaxel	Wnt	Ib
Solid tumors/general	Bronticuzumab^e^	Notch	Ia
	OMP-305B83	DLL4/VEGF	Ia
	Napabucasin^c^	STAT	II
	Amcasertib^d^	Broad spectrum, incl. Nanog	I/II
Urologic malignancy	Amcasertib^d^	Broad spectrum, incl. Nanog	II
T cell lymphoma, relapsed/refractory	Duvelisib^b^	PI3K Delta/PI3K Gamma	I/Ib

CLL = chronic lymphocytic leukemia; FCR = fiudarabine, cyclophosphamide, and rituximab; iNHL = indolent non-Hodgkin’s lymphoma; PI3K = phosphoinositide-3-kinase; SOC = standard of care

a Also known as OMP-18R5

b IPI-145

c BB608, BBI608, BBI-608

d BBI-503

e Anti-Notch 1, OMP-52M

f VS-6063

g Anti-DLL4, OMP-21M18

h FZD8-Fc, OMP-54F2851

i Anti-Notch 2, OMP-59R5.

Data source: Ajani et al. (2015).^57^

**Table 3:** Markers of cancer stem cells according to tumor tissue origin

Cancer type	Marker	Reference
Brain	CD133, CD90	Singh et al., 2003^220^ He et al., 2012^221^
Breast	ESA, CD44, CD24, ALDH	Al-Hajj M et al., 2003^31^ Ginestier et al., 2007^222^
Colorectal	ESA, CD133, CD166, CD44, CD24, ALDH	Vaiopoulos et al., 2012^223^ Cherciu et al., 2014^224,225^ Huang et al., 2009^226^
Endometrial	CD133	Rutella et al., 2009^227^
Gastric	CD44	Takaishi et al., 2009^228^ Zhao et al., 2015^79^
Head and neck	CD44, CD24, ALDH	Han J et al., 2014^229^
Hematologic	CD34, CD38	Lapidot et al., 1994^27^
Leukemia	CD34, CD38, CD47, CCL-1, CD96, TIM3, CD32, CD25	Bonnet and Dick, 1997^28^ Majeti et al., 2009^230^ van Rhenen et al., 2007^231^ Hosen et al., 2007^232^ Jan et al.,2011^233^ Saito et al., 2010^234^
Liver	ESA, CD133, CD90, CD44, CD24, ALDH	Yamashita and Wang, 2013^235^ Ma et al., 2008^236^
Lung	CD133, CD44, CD90, ABCG2 or CXCR4	Alamgeer et al., 2013^237^ Donnenberg et al., 2007^238^ Bertolini et al., 2009^239^
Melanoma	ALDH	Boonyaratanakornkit et al., 2010^240^
Ovarian	CD44^+^, c-Kit	Zhang et al., 2008^240^
Pancreatic	ESA, CD44, CD24, CXCR4, ALDH	Li et al., 2014^242^ Herman et al., 2007^162^
Prostate	integrin α2β1, CD44^+^	Collings et al., 2005^39^

ALDH = aldehyde dehydrogenase; CLL = C-type lectin-like molecule-1; ESA = epithelial-specific antigen.
